# Treatment outcome of pneumonia and its associated factors among pediatric patients admitted to Hiwot Fana Comprehensive Specialized University Hospital, Eastern Ethiopia

**DOI:** 10.3389/fped.2024.1296193

**Published:** 2024-04-26

**Authors:** Gebremariam Adbela, Hanan Abdurahman, Saba Hailu, Mulualem Keneni, Ahmed Mohammed, Fitsum Weldegebreal

**Affiliations:** ^1^ School of Medicine, College of Health and Medical Sciences, Haramaya University, Harar, Ethiopia; ^2^ School of Public Health, College of Health and Medical Sciences, Haramaya University, Harar, Ethiopia; ^3^ School of Nursing and Midwifery, College of Health and Medical Sciences, Haramaya University, Harar, Ethiopia; ^4^ School of Medical Laboratory Sciences, College of Health and Medical Sciences, Haramaya University, Harar, Ethiopia; ^5^ Laboratory Bacteriology Research, Faculty of Medicine and Health Sciences, Ghent University, De Pintelaan, Ghent, Belgium

**Keywords:** treatment outcome, childhood, pneumonia, Eastern Ethiopia, pediatrics

## Abstract

**Background:**

Pneumonia is the leading cause of morbidity and mortality among children worldwide. Despite its substantial impact, there exists a dearth of evidence regarding treatment outcomes and related factors, particularly within the Ethiopian context. This study endeavors to address these critical gaps by examining the treatment outcome of pneumonia among pediatric patients hospitalized in the Hiwot Fana Comprehensive Specialized University Hospital.

**Method:**

A facility-based cross-sectional study was conducted on 204 children (≤14 years of age) diagnosed with pneumonia and admitted to the Hiwot Fana Comprehensive Specialized University Hospital. An interview using a structured questionnaire accompanied by a review of medical records was used to collect data from the parents/guardians. A binary logistic regression model with an adjusted odds ratio (AOR) and a 95% confidence interval (CI) was used to identify the associated factors with the outcome variable. Statistical significance was set at *P* < 0.05 in the multivariable analysis.

**Result:**

Among the 204 children (≤14 years) included in the study, 119 (93.6%, 95% CI: 90.2–96.9) patients with pneumonia survived whereas 13 (6.4%, 95% CI: 3.1–9.7) died. Multivariable logistic regression analysis, after adjustments for potential confounders, revealed that children who had malnutrition (AOR = 3.5, 95% CI: 2.37–12.44), were unvaccinated (AOR =  3.41, 95% CI: 2.25–11.87), had altered mental states during admission (AOR = 4.49, 95% CI: 2.28–17.85), and had complicated types of pneumonia (AOR = 5.70, 95% CI: 2.98–15.09) were independently associated with mortality.

**Conclusion:**

Poor treatment outcome was 6.4% among pediatric patients admitted with pneumonia in this study setting. Being unvaccinated, malnourished, and admitted with a complicated type of pneumonia as well as having altered consciousness at the time of admission were significantly associated with poor treatment outcomes. These findings underscore the critical need to prioritize preventative measures against malnutrition and unvaccinated status in children. Early identification of such children and proper interventions are imperative to reducing such outcomes, particularly in resource-constrained settings.

## Introduction

Pneumonia is one of the most serious infections affecting the lung alveolar air spaces (1). Based on the setting of occurrence, pneumonia is classified as community-acquired and hospital-acquired (nosocomial) pneumonia. Community-acquired pneumonia is an infection that initiates outside the hospital or is diagnosed within 48 h after admission to the hospital (2). A very large number of pathogens, including bacteria, viruses, and fungi, can cause childhood pneumonia. *Streptococcus pneumoniae, Haemophilus influenzae* type B, and respiratory syncytial virus (RSV) are the main pathogens associated with childhood pneumonia with the first two being vaccine-preventable causes (3). *Streptococcus pneumonia* causes 18% of severe cases of pneumonia and 33% of deaths globally and *Haemophilus influenzae* type B is responsible for 4% of severe episodes and 16% of deaths (4).

Pneumonia remains the leading cause of morbidity and mortality among children worldwide (5, 6). There are 120 million incidences of pneumonia in a year; sub-Saharan African countries followed by Southeast East Asian countries share the highest burden of cases among children under 5 years of age (4). The African region is home to 20% of the world's population of children under 5 years of age. It comprises about 45% of global under-5 mortality, and 50% of worldwide deaths are from pneumonia. On the contrary, less than 2% of this mortality occurred in European countries and less than 3% occurred in the US (7). Nearly half (49%) of total deaths from pneumonia worldwide in 2015 were concentrated in five countries of Sub-Saharan Africa and Asia: India, Nigeria, Pakistan, the Democratic Republic of the Congo, and Ethiopia (8).

Evidence revealed that the leading risk factors for childhood pneumonia are suboptimal breastfeeding, under nutrition, lack of immunization, crowding, indoor air pollution, low socioeconomic status, zinc deficiency, and low birth weight, which have a role to play in increasing children's susceptibility to pneumonia (7, 9, 10).

Evidence from several retrospective cohort studies indicated that early life exposure (intrauterine and post-natal exposure) to air pollution and indoor environmental factors were significantly associated with pneumonia development, supporting the hypothesis of “Preconceptional and Fetal Origin of Childhood Pneumonia” (11, 12).

The burden of childhood pneumonia is high in Ethiopia children due to limited coverage and affordability of effective preventive interventions like immunization (13). It is estimated that 3,370,000 children in Ethiopia encounter pneumonia which contributes to 18% of all under-five deaths, it kills over 40,000 children every year (14). According to a study conducted in Jimma, Ethiopia, the prevalence of pneumonia among children under the age of 5 who visit hospitals was 28.1%, of which 10% was of severe pneumonia (15). Approximately only 31% of children with symptoms of pneumonia have utilized healthcare in Ethiopia (16). Currently, the health policies in Ethiopia are focusing on reducing mortality associated with pneumonia among children under the age of 5 by scaling up immunization and case management of pneumonia (17).

Case management is a cornerstone of pneumonia control strategies and requires early identification and treatment (18). Treatment outcomes might be affected by resistance, the initial severity of the disease, and the presence of co-morbid conditions. Studying predictors for the treatment outcome, childhood pneumonia has a significant role in designing appropriate interventions and reducing the incidence of treatment failure among patients with pneumonia. Studies assessing the treatment outcome of pneumonia among children in Ethiopia are limited. Hence, this study aims to fill the existing practical gaps by assessing the treatment outcome of pneumonia among pediatric patients hospitalized in the Hiwot Fana Comprehensive Specialized University Hospital (HFCSUH).

## Materials and methods

### Study setting and design

A facility-based cross-sectional study was conducted among pediatric patients hospitalized with pneumonia at HFCSUH, Harar, Ethiopia, from 15 October 2022 to 15 January2023. The Hiwot Fana Comprehensive Specialized University Hospital is situated in the eastern part of Ethiopia 525 km away from Addis Ababa and it is among the pioneering teaching university hospitals in Ethiopia. The hospital offers a wide range of specialties, including pediatrics, surgery, gynecology, oncology, and psychiatry. The pediatric ward accommodates a total of 63 beds [six in the pediatric intensive care unit (PICU), 17 in the pediatric ward, 23 in the emergency ward, and 15 in the nutritional rehabilitation unit (NRU)]. The Hospital admits about 3,500 pediatric patients per year.

### Population and sampling technique

The sample size was calculated using a single population sample calculation formula, considering the prevalence of death associated with pneumonia among pediatric patients from the previous study was 16% (13), confidence level of 95%, a 5% margin of error, and a 10% non-response rate resulted in a final sample size of 204. Patients with age ≤14 years admitted with the clinical diagnosis of pneumonia in a pediatric ward of HFCSUH were included consecutively until the determined sample size was reached. Pediatric patients who developed pneumonia after 48 h of admission, patients referred to other healthcare facilities, patients who died before initiation of treatment, and those aged less than 1 month were excluded from the study.

### Data collection tool and procedure

Data were collected using a pretested structured questionnaire. The questionnaire was developed after reviewing the relevant literature extensively. Face-to-face interviews and simultaneously chart reviews were conducted to extract additional relevant data from the medical records. Two pediatric residents were recruited as data collectors and a senior pediatrician as a supervisor and received two days of training on the content of the data collection tool.

### Method of data analysis

Data were entered into the software Epi-data version 4.6 and then exported for further analysis into the SPSS version 26.0 software. Descriptive statistics such as frequencies, means, median, and standard deviations were calculated to summarize the explanatory variables and treatment outcomes of pneumonia cases. Bivariate and multivariable analyses were conducted to determine the association between predictive factors and treatment outcomes of pneumonia. Associations were described using an adjusted odds ratio (AOR) and with a 95% confidence interval (CI). A *p*-value less than 0.05 was considered statistically significant.

### Measurements

According to the World Health Organization (WHO) guideline, pneumonia is diagnosed in children when they have a cough or difficulty breathing along with lower chest wall in drawing or age-appropriate tachypnea (age-specific respiratory rates become ≥60/min in infants aged <2 months, ≥50/min in infants aged 2 to <12 months, and ≥40/min in children aged 12–59 months). Chest x-rays, blood, and other tests done support the diagnosis (3, 19, 20). Complicated pneumonia was diagnosed by combinations of local complications such as parapneumonic effusion, empyema, necrotizing pneumonia, and lung abscess and systemic complications such as bacteremia, metastatic infection, multiorgan failure, acute respiratory distress syndrome, and disseminated intravascular coagulation (21).

#### Nutritional status

Nutritional status was computed based on weight-for-age *Z* scores (WAZ), height-for-age *Z* scores (HAZ), and weight-for-height *Z* scores (WHZ) by the least mean squares method and the 2000 Centers for Disease Control and Prevention growth reference curves (22). Weight was measured using a weighing scale (salter-type baby weighing scale for infants and those who could not stand) and a digital electric weighing scale when the child was completely undressed and rounded to the nearest 0.1 kg. The body length/height was measured using a stadiometer and length measuring board and recorded in centimeters.

#### Immunization status

A child health card was used to assess the immunization status of children. Appropriate history about immunization using a checklist was used for caretakers who did not have immunization cards at the time of data collection. Children who had received some vaccinations expected for their age were labeled as partially vaccinated, and those that had never been vaccinated at all were labeled as not vaccinated (23).

### Sample collection and laboratory procedures

The human immunodeficiency virus (HIV) rapid test was done using finger prick blood samples in the wards by a qualified counselor based on the manufacturer's instructions following the standard operating procedures. A rapid antibody test was used to screen HIV infection in all patients aged above 18 months.

Total white blood cell count, differential, and hemoglobin were determined after taking 2 ml venous blood on an ethylenediamine acetic acid (EDTA) tube and then measured on an automated machine (Hematologic analyzer machine) that displays the results of complete blood cell count after analyzing the blood sample (24).

### X-ray interpretation

Standard posterior–anterior or anterior–posterior chest x-rays were taken and the finding was interpreted independently by one radiologist and two pediatricians following a WHO-designed x-ray interpretation protocol. Evidence of consolidation and/or pleural effusion and/or interstitial infiltration was defined as pneumonia (25).

### Operational definition

#### Treatment outcome

The result obtained after the treatment. It might be *improved* or *died* (26).

#### Good treatment outcome

It represents the discharge from the hospital on the grounds of clinical improvement (26, 27).

#### Poor treatment outcome

It is defined as the occurrence of death (26, 27).

### Ethical consideration

The study was carried out following the Declaration of Helsinki, and ethical approval was obtained from the Haramaya University College of Health and Medical Sciences Institutional Health Research Ethics Review Committee (IHRERC). Official letters of cooperation were submitted to HFCSUH and concerned bodies to obtain their cooperation and consent in facilitating the study. The respondents were informed of their right to refuse or decline participation in the study at any time and that refusing to participate in the study will not affect them. Informed consent was obtained before the study from the subject and parent(s) or guardian(s). Participants’ confidentiality of information was assured by excluding names and identifiers in the questionnaire.

## Results

### Sociodemographic characteristics

A total of 204 cases were included in the study. More than one-third (35.3%) of the patients were found in the age range of 1–3 years and 117 (57.4%) were boys. The majority of the caregivers of the children have no formal education and 122 (59.8%) caregivers were housewives (Table 1).

**Table 1:** Sociodemographic characteristics of patients with pneumonia in the pediatric ward, HFCSUH, Harar, Eastern Ethiopia, 2023 (*N* = 204).

Variables	Treatment outcome		Frequency	Percentage
	Improved	Died		
Age				
1 month to <1 year	61	7	68	33.3
1 to 3 years	69	3	72	35.3
3 to <6 years	34	2	36	17.6
≥6 years	27	1	28	13.7
Sex				
Male	110	7	117	57.4
Female	81	6	87	42.6
Educational status of the caregiver				
No formal education	114	8	122	59.8
Primary education	54	3	57	27.9
Secondary education	15	2	17	8.3
College and above	8	—	8	3.9
Occupation of caregiver				
Housewife	117	5	122	59.8
Merchant	45	7	52	25.5
Farmer	16	1	17	8.3
Government employee	13	—	13	6.4

### Medical and environmental factors

Among the selected pediatric patients with pneumonia, 136 (66.7%) were vaccinated. One-fourth of the study participants were malnourished, and 97 (47.5%) of them were not exclusively breast fed. More than one-third of pneumonia patients had comorbidity, and anemia was the most common (39.4%) condition followed by heart failure (16.9%). A total of 102 (50.5%) of the children's weights were in the range of 6–10 kg. History of exposure to cigarettes and no separate kitchen in the house was reported by 111 (54.4%) and 67 (32.8%) caregivers, respectively (Table 2).

**Table 2:** Medical and environment-related factors of patients with pneumonia in the pediatric ward, HFCSUH, Harar, Eastern Ethiopia, 2023 (*N* = 204).

Medical and environmental variables	Treatment outcome		Frequency	Percentage
	Improved	Died		
Immunization status				
Vaccinated	131	5	136	66.7
Not vaccinated	60	8	68	33.3
Nutritional status				
Malnourished	45	7	52	25.5
No malnourishment	146	6	152	74.5
Exclusively breast fed				
Yes	102	5	107	52.5
No	89	8	97	47.5
Comorbidity				
HAAD/asthma	9	—	9	12.7
Anemia	25	3	28	39.4
CHF/CHD	10	2	12	16.9
Measles	10	1	11	15.5
Others	9	2	11	15.5
Weight (kg)				
1–5	22	3	25	11.8
6–10	96	6	102	50.5
11–15	47	3	50	23.5
≥16	26	1	27	14.2
Exposed to cigarette smoke				
Yes	104	7	111	54.4
No	87	6	93	45.6
Separate kitchen				
Yes	131	6	137	67.2
No	60	7	67	32.8

HAAD, hyperactive airway disease; CHF, congestive heart failure; CHD, congenital heart defects.

### Clinical-related factors of the study population

At the time of admission, about 166 (81.4%) of the patients were alert, 70 (34.3%) had high-grade fever, 177 (86.7%) had fast breathing with respiratory distress, and 149 (73%) of them had tachycardia. More than half (55.4%) of patients were given antibiotics prior to admission and 70 (34.3%) of them had previous history of pneumonia. In total, 157 (77%) patients were admitted with the diagnosis of non-complicated pneumonia (Table 3).

**Table 3:** Clinic-related factors of patients with pneumonia in the pediatric ward, HFCSUH, Harar, Eastern Ethiopia, 2023 (*N* = 204).

Clinical variable	Treatment outcome		Frequency	Percentage
	Improved	Died		
Level of consciousness				
Alert	160	6	166	81.4
Impaired consciousness	31	7	38	18.6
Temperature				
Normal	53	3	56	27.5
Low-grade fever	74	4	78	38.2
High-grade fever	64	6	70	34.3
Respiration				
Normal with respiratory distress	2	2	4	2
Tachypnea	22	1	23	11.3
Tachypnea with respiratory distress	167	10	177	86.7
Pulse rate				
Normal range	53	2	55	27
Tachycardic	138	11	149	73
Oxygen saturation				
≥89%	83	7	91	44.6
≥90%	108	6	113	55.4
Seizure				
Yes	8	2	10	4.9
No	183	11	194	95.1
Central cyanosis				
Yes	28	5	33	16.2
No	163	8	171	83.8
Chest auscultation				
No finding	51	—	51	25
Crepitation	76	7	83	40.7
Bronchial breath sound	10	1	11	5.4
Decreased or absent air entry	35	3	38	18.6
Wheezing and crepitation	16	2	18	8.8
Others	3	–	3	1.5
Severity of pneumonia				
Non-complicated pneumonia	152	5	157	77
Complicated pneumonia	39	8	47	23
Type of complication				
Empyema/parapneumonic effusion	33	2	35	74.6
Lung abscess	1	1	2	4.2
Pneumothorax	1	1	2	4.2
Empyema and pericarditis	1	1	2	6.2
Meningitis	2	2	4	8.6
Others	1	1	2	4.2
Duration of illness				
<24 h	9	—	9	4.4
24 to <72 h	48	3	51	25
3–7 days	62	3	65	31.9
>7 days	72	7	79	38.7
Antibiotics given before admission				
Yes	103	10	113	55.4
No	88	3	91	44.6
Previous history of pneumonia				
Yes	62	8	70	34.3
No	129	5	134	65.7

### Specific complications of pneumonia among the study population

**Figure 1:**
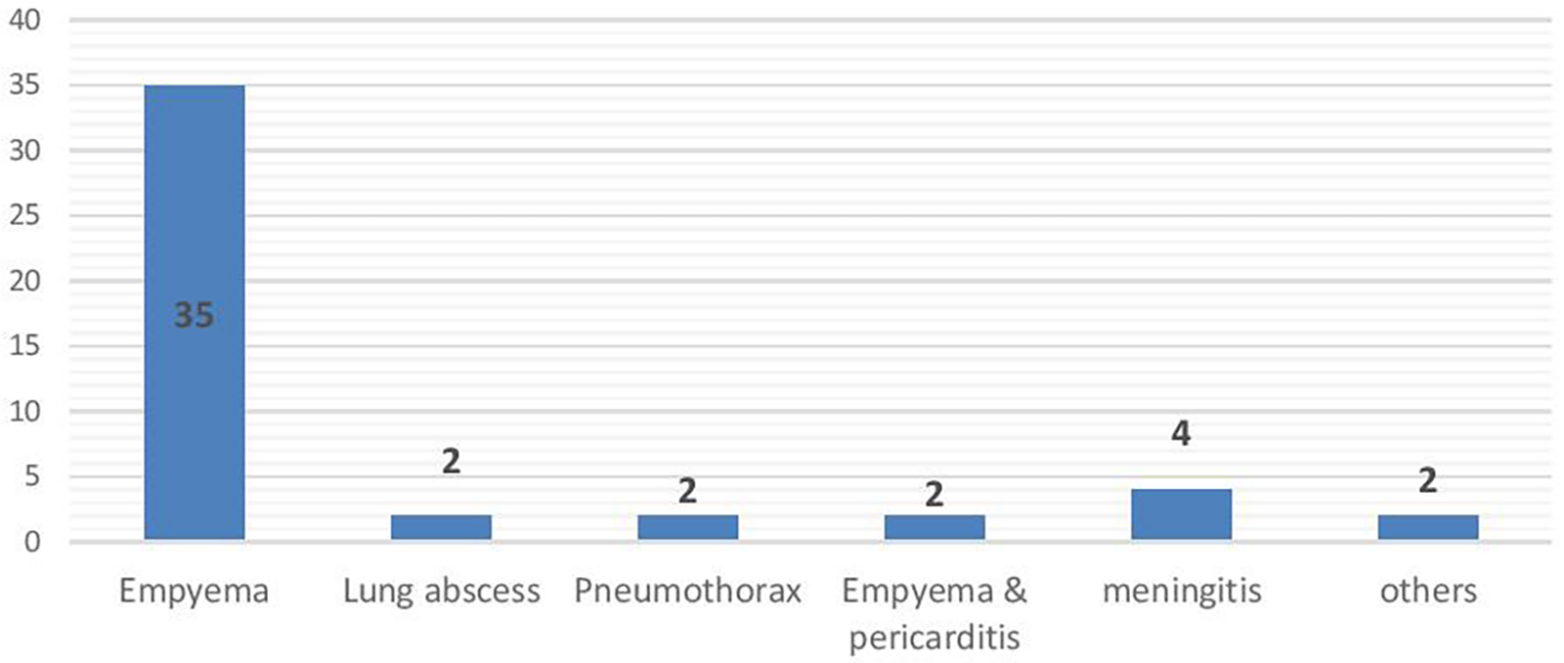
Specific complications of pneumonia among patients in the pediatric ward of HFCSUH, Harar, Eastern Ethiopia, 2023 (*N* = 204).

Among the 204 children, 47 (23%) them had complications of pneumonia. Empyema was the most common complication among 35 (74.5%) pediatric patients followed by meningitis among 4 (8.5%) patients (Figure 1).

### Laboratory and radiology-related findings

The majority of the study population, 166 (81.4%) patients, had a normal range of lymphocyte counts and nearly half of them, 98 (48%) patients, had a normal range of total white blood cells. Three of the patients were reactive for HIV serology tests. A total of 119 (58.3%) of them had chest x-ray findings, and 41 (34.5%) of the patients had homogenous opacity in chest x-rays. Approximately 53 (26%) of the patients had chest ultrasound findings, and about 28 (13.7%) of them had empyema (Table 4).

**Table 4:** Laboratory and radiology-related factors of patients with pneumonia in the pediatric ward, HFCSUH, Harar, Eastern Ethiopia, 2023 (*N* = 204).

Laboratory and radiology findings	Treatment outcome		Frequency	Percentage
	Improved	Died		
WBC count (cell/mic Lt)				
Normal for his/her age	95	3	98	48
Low for his/her age	15	3	18	8.8
High for his/her age	81	7	88	43.2
Neutrophil (%)				
Normal for his/her age	100	4	104	51
Low for his/her age	16	2	18	8.8
High for his/her age	75	7	82	40.1
Lymphocyte (%)				
Normal for his/her age	162	4	166	81.4
Low for his/her age	19	7	26	12.7
High for his/her age	10	2	12	5.9
HIV serology test				
Reactive	1	2	3	1.5
Non-reactive	109	11	120	58.8
Chest x-ray finding				
Yes	106	11	117	57.4
No	85	2	87	42.6
Chest x-ray specific finding				
Consolidation	24	3	27	22.7
Infiltration	32	4	36	30.2
Homogenous opacity	37	4	41	34.5
Collapse	3	2	5	4.2
Others	10	—	10	8.4
Chest ultrasound				
Not done	144	7	151	74
Empyema	26	2	28	13.7
Simple effusion	7	—	7	3.4
Consolidation	14	4	18	8.8

### Antibiotics and admission-related characteristics

The most commonly prescribed drug was ceftriaxone, for 64 (33.3%) patients, followed by the combination of ceftriaxone and vancomycin, among 44 (22.9%) children. The initial antibiotics were revised among 58 (28.4%) patients. Regarding the duration of hospitalization, 60 (29.4%) children stayed in the hospital for more than 7 days (Table 5).

**Table 5:** Antibiotics and treatment given to patients with pneumonia in the pediatric ward, HFCSUH, Harar, Eastern Ethiopia 2023 (*N* = 204).

Antibiotics related variables	Treatment outcome		Frequency	Percentage
	Improved	Died		
Antibiotics given on admission				
Ceftriaxone	62	2	64	33.3
Ceftriaxone + azithromycin	28	1	29	15.1
Ampicillin + gentamycin	38	1	39	20.3
Ceftriaxone** **+** **vancomycin	37	7	44	22.9
Ceftriaxone + ampicillin	7	1	8	4.2
Others	7	1	8	4.2
Initial antibiotic revised				
Yes	51	7	58	28.4
No	140	6	146	71.6
Duration of hospitalization				
24 to <72 h	52	2	54	26.5
3–7 days	87	3	90	44.1
>7 days	52	8	60	29.4

### Treatment outcome of pneumonia

Out of 204 children admitted with pneumonia, 13 children (6.4%) (95% CI: 95% CI: 3.1–9.7) died due to pneumonia, while 119 children (93.6%) (95% CI: 90.2–96.9) were cured and discharged.

Seven of the 13 deaths occurred among children less than 1 year of age. Eight of the deaths occurred due to complicated pneumonia and the remaining five deaths were due to non-complicated pneumonia. Of the total deaths, eight children were not vaccinated, seven had Malnutrition and eight were not exclusively breastfed.

### Factors associated with pneumonia treatment outcome

In multivariable logistic regression analysis, it had been shown that children that were unvaccinated, had malnutrition, or the presence of complicated pneumonia, or altered consciousness at the time of admission were significantly associated with poor treatment outcomes of pneumonia at a *P*-valve less than 0.05.

Children with malnutrition were 3.5 times (AOR = 3.5, 95% CI: 2.37–12.44) more likely to die with pneumonia than children who had normal nutritional status. Children who were not vaccinated had 3.4 times (AOR = 3.41, 95% CI: 2.25–11.87) higher odds of dying due to pneumonia compared with their counterparts. Having altered mentation at the time of admission was 4.5 times (AOR = 4.49, 95% CI: 2.28–17.85) more likely to lead to development of poor treatment outcomes of pneumonia than counterparts. Similarly, children having complicated pneumonia had 5.7-fold (AOR = 5.70, 95% CI: 2.98–15.09) higher odds of having poor treatment outcomes for pneumonia than children who had non-complicated pneumonia (Table 6).

**Table 6:** Factors associated with treatment outcome of pneumonia in the pediatric ward, HFCSUH, Harar, Eastern Ethiopia, 2023 (*N* = 204).

Exposure variables	Treatment outcome		COR (95% CI)	AOR (95% CI)	*P*-value
	Died *N* = 13	Improved *N* = 191			
Nutritional status					
Non-malnourished	6 (3.9%)	146 (96.1%)	1	1	0.020*
Malnourished	7 (13.5%)	45 (86.5%)	3.78 (1.21–11.84)	3.5 (2.37–12.44)	
Vaccination status					
Vaccinated	5 (3.7%)	131 (96.3%)	1	1	0.026*
Not vaccinated	8 (12.8%)	60 (88.2%)	3.49 (1.11–11.13)	3.41 (2.25–11.87)	
Level of consciousness					
Alert	6 (3.6%)	160 (96.4%)	1	1	0.003**
Impaired	7 (18.4%)	31 (81.6%)	6.02 (2.52–16.19)	4.49 (2.28–17.85)	
Central cyanosis					
No	8 (4.7%)	163 (95.3%)	1	1	0.93
Yes	5 (15.2%)	28 (84.8%)	3.64 (1.11–11.93)	3.63 (0.81–16.35)	
Severity of pneumonia					
Non-complicated	5 (3.2%)	152 (96.8%)	1	1	0.035*
Complicated	8 (17.0%)	39 (83.0%)	6.24 (1.93–10.12)	5.70 (2.98–15.09)	
Comorbidity					
No	5 (3.8%)	128 (96.2%)	1	1	0.29
Yes	8 (11.3%)	63 (88.7%)	3.25 (1.02–10.34)	2.28 (0.49–10.63)	
Previous history of pneumonia/LRTI					
No	5 (3.7%)	129 (96.3%)	1	1	0.56
Yes	8 (11.4%)	62 (88.6%)	3.33 (1.05–10.59)	1.53 (0.36–6.38)	
Antibiotics revision					
No	6 (4.1%)	140 (95.9%)	1	1	0.91
Yes	7 (12.1%)	51 (87.9%)	3.20 (1.03–9.98)	1.09 (0.26–4.62)	
Oxygen saturation					
≥90%	6 (5.3%)	107 (94.7%)	1	1	0.32
≤89%	7 (6.2%)	84 (93.8%)	2.01 (0.66–6.59)	1.24 (1.93–13.12)	
Exclusive breastfeeding					
Yes	5 (4.7%)	102 (95.3%)	1	1	0.61
No	8 (8.2%)	89 (91.8%)	1.83 (0.58–5.81)	1.4 (1.7–9.59)	

COR, crude odds ratio; LRTI, lower respiratory tract infection.

* *p* ≤ 0.05.

** *p* ≤ 0.003.

## Discussion

Globally, pneumonia is the leading cause of morbidity and mortality among children (5, 6), with the highest burden of disease in sub-Saharan Africa and Southeast Asian countries (4, 8). Identifying the predictors for treatment outcomes of childhood pneumonia is a significant input to reducing child mortality. This study was conducted to assess the treatment outcome of pneumonia and its associated factors among pediatric patients admitted to the Hiwot Fana Comprehensive Specialized University Hospital, Eastern Ethiopia. About 6.4% of pediatric patients admitted with pneumonia had poor treatment outcomes, with an increased risk of death to unvaccinated children, malnourished children, those with altered consciousness at the time of admission, and those having a complicated type of pneumonia.

In this study, the case fatality rate of pneumonia among hospitalized children was 6.4%. This study was comparable with studies conducted in the Philippines (4.7%) (28), Rabat, Morocco (4%) (29), Nekemt Referral Hospital, Ethiopia (5.9%) (30), and Tikur Anbessa Specialized Hospital, Ethiopia (7.7%) (26). Conversely, mortality in this study was higher than in the study conducted in China (2.4%) (31). The discrepancy can be explained by differences in case management interventions for childhood pneumonia in developing countries and the quality of the healthcare system in high-income countries (32). However, it is lower than studies conducted in New Delhi, India (10.5%) (33) and Khartoum, Sudan (16.9%) (34). This might be due to the differences in the study population since the aforementioned studies were conducted among children under the age of 5 years.

The present findings indicate that children with malnutrition had a 3.5 times higher risk of dying than children with pneumonia and normal nutritional status. This study is consistent with studies conducted in the Philippines (28), Tanzania (24), and Ethiopia (13, 26). Malnourished children are known to be immunocompromised, particularly susceptible to infections causing severe diseases, and associated with poor outcomes. Studies showed that malnutrition is a major risk factor for severe pneumonia and is associated with mortality (35, 36). Early treatment of malnourished children with pneumonia is critical to improve their survival.

In this study, unimmunized children were 3.4 times more likely to die than immunized children diagnosed with pneumonia. This study is supported by studies conducted in Morocco (29), Bangladesh (37), Tikur Anbessa Specialized Hospital, Ethiopia (26), and Jimma University Specialized Hospital, Ethiopia (13). This might be due to suboptimal coverage of the pneumococcal conjugate vaccine (PCV) in the study area. A population-based longitudinal study conducted in eastern Ethiopia showed only 39% of the children were fully vaccinated and the total coverage of PCV was 66% in the area (38). An intervention targeting anti-pneumococcal and Hib vaccines will undoubtedly result in a significant decrease in pneumonia-related mortality among children.

In the present study, children with impaired consciousness on admission were 4.5 times more likely to have poor treatment outcome of pneumonia than alert children. This finding is in line with studies conducted in India (39) and Morocco (29). Altered consciousness is an indicator of the level or severity of the disease that ultimately defines the treatment outcome.

Furthermore, this study found that children with complicated pneumonia were six times more likely to have poor treatment outcomes for pneumonia, compared with children with non-complicated pneumonia. This might be due to the low effectiveness of the treatment regimen due to antibiotic resistance leading to complications that affect the vital organs of the patients. Clinical trials and other interventions should be employed to provide definitive antibiotics for patients with severe pneumonia.

### Limitations of the study

The study does not establish a definitive cause-and-effect relationship among variables. Moreover, it is a single-facility-based study; caution should be taken in extrapolating the results, potentially underestimating the true burden of pneumonia. In addition, data on vaccination status were gathered through verbal reports from guardians, introducing the possibility of recall bias. Furthermore, due to the small sample size, subgroup analysis could not be conducted, emphasizing the necessity for future studies with larger sample sizes to enable more robust analyses.

## Conclusion

Poor treatment outcome was derived for 6.4% of the pediatric patients admitted with pneumonia in this study setting. Factors associated with poor treatment outcome included being unvaccinated, malnourished, and admitted with severe pneumonia as well as having altered consciousness at the time of admission. Early identification of such high-risk children and implementation of proper interventions are imperative to reducing such poor treatment outcomes, particularly in resource-constrained settings. Continued surveillance is essential for identifying unvaccinated children in the study area. In addition, multicenter studies have the potential to explore other environmental and personal factors contributing to poor treatment outcomes, providing further insights for improving pediatric pneumonia management.

## Data Availability

The original contributions presented in the study are included in the article/Supplementary Material; further inquiries can be directed to the corresponding author.

## References

[1] Markos Y Dadi AF Demisse AG Habitu YA Derseh BT Debalkie G. Determinants of under-five pneumonia at Gondar University Hospital, Northwest Ethiopia: an unmatched case-control study. J Environ Public Health. (2019) 2019:8. 10.1155/2019/9790216 PMC6778888 31662768

[2] Porth C. Pathophysiology: Concepts of Altered Health States. Philadelphia: Lippincott Williams & Wilkins (2005).

[3] Zar H Madhi S Aston S Gordon S. Pneumonia in low and middle income countries: progress and challenges. Thorax. (2013) 68(11):1052–6. 10.1136/thoraxjnl-2013-204247 23956020 PMC3960724

[4] Walker CLF Rudan I Liu L Nair H Theodoratou E Bhutta ZA Global burden of childhood pneumonia and diarrhoea. Lancet. (2013) 381(9875):1405–16. 10.1016/S0140-6736(13)60222-6 23582727 PMC7159282

[5] Williams DJ Creech CB Walter EB Martin JM Gerber JS Newland JG Short- vs standard-course outpatient antibiotic therapy for community-acquired pneumonia in children: the SCOUT-CAP randomized clinical trial. JAMA Pediatr. (2022) 176(3):253–61. 10.1001/jamapediatrics.2021.5547 35040920 PMC8767493

[6] Dean P Florin TA. Factors associated with pneumonia severity in children: a systematic review. J Pediatric Infect Dis Soc. (2018) 7(4):323–34. 10.1093/Jpids/Piy046 29850828 PMC6454831

[7] Rudan I Boschi-Pinto C Biloglav Z Mulholland K Campbell H. Epidemiology and etiology of childhood pneumonia. Bull World Health Organ. (2008) 86(5):408–16. 10.2471/BLT.07.048769 18545744 PMC2647437

[8] McAllister DA Liu L Shi T Chu Y Reed C Burrows J Global, regional, and national estimates of pneumonia morbidity and mortality in children younger than 5 years between 2000 and 2015: a systematic analysis. Lancet Glob Health. (2019) 7(1):e47–57. 10.1016/S2214-109X(18)30408-X 30497986 PMC6293057

[9] Goyal JP Kumar P Mukherjee A Das RR Bhat JI Ratageri V Risk Factors for the Development of Pneumonia and Severe Pneumonia in Children. Indian Pediatr. (2021) 58(11):1036–9. PMID: 34837363

[10] Wonodi CB Deloria-Knoll M Feikin DR DeLuca AN Driscoll AJ Moïsi JC Evaluation of risk factors for severe pneumonia in children: the pneumonia etiology research for child health study. Clin Infect Dis. (2012) 54(suppl_2):S124–31. 10.1093/Cid/Cir1067 22403226 PMC3297552

[11] Lu C Yang W Wang F Li B Liu Z Liao H. Effects of intrauterine and post-natal exposure to air pollution on children’s pneumonia: key roles in different particulate matters exposure during critical time windows. J Hazard Mater. (2023) 457:131837. 10.1016/j.jhazmat.2023.131837 37329598

[12] Lu C Yang W Liu Z Liao H Li Q Liu Q. Effect of preconceptional, prenatal and postnatal exposure to home environmental factors on childhood pneumonia: a key role in early life exposure. Environ Res. (2022) 214:114098. 10.1016/j.envres.2022.114098 35981613

[13] Tegenu K Geleto G Tilahun D Bayana E Bereke B. Severe pneumonia: treatment outcome and its determinant factors among under-five patients, Jimma, Ethiopia. SAGE Open Med. (2022) 10:20503121221078445. 10.1177/20503121221078445 35223030 PMC8873968

[14] Liu L Oza S Hogan D Perin J Rudan I Lawn JE Global, regional, and national causes of child mortality in 2000–13, with projections to inform post-2015 priorities: an updated systematic analysis. Lancet. (2015) 385(9966):430–40. 10.1016/S0140-6736(14)61698-6 25280870

[15] Lema K Murugan R Tachbele E Negussie B. Prevalence and associated factors of pneumonia among under-five children at public hospitals in Jimma zone, south west of Ethiopia, 2018. J Pulmonol Clin Res. (2018) 2(1):25–31.

[16] CSA I. Central Statistical Agency. Ethiopian Demographic and Health Survey 2016. Addis Ababa: CSA and ICF (2016).

[17] Alebachew A Hatt L Kukla M. Monitoring and evaluating progress towards universal health coverage in Ethiopia. PLoS Med. (2014) 11(9):e1001696. 10.1371/journal.pmed.1001696 25244146 PMC4171462

[18] Revised WHO Classification and Treatment of Pneumonia in Children at Health Facilities: Evidence Summaries. Geneva: World Health Organization (2014). PMID: 25535631

[19] Ebell MH. Clinical diagnosis of pneumonia in children. Am Fam Physician. (2010) 82(2):192–3. 20642275

[20] Ashraf H Chisti MJ Alam NH. Treatment of Childhood Pneumonia in Developing Countries. Bangladesh: INTECH Open Access Publisher (2010).

[21] de Benedictis FM Kerem E Chang AB Colin AA Zar HJ Bush A. Complicated pneumonia in children. Lancet. (2020) 396(10253):786–98. 10.1016/S0140-6736(20)31550-6 32919518

[22] Kuczmarski RJ Ogden CL Guo SS Grummer-Strawn LM Flegal KM Mei Z 2000 CDC growth charts for the United States: methods and development. Vital Health Stat. (2002) (246):1–190. PMID: . 12043359

[23] Kiconco G Turyasiima M Ndamira A Yamile OA Egesa WI Ndiwimana M Prevalence and associated factors of pneumonia among under-fives with acute respiratory symptoms: a cross sectional study at a teaching hospital in Bushenyi district, Western Uganda. Afr Health Sci. (2021) 21(4):1701–10. 10.4314/ahs.v21i4.25 35283986 PMC8889805

[24] Muro RP Masoza TS Kasanga G Kayange N Kidenya BR. Predictors and outcome of first line treatment failure among under-five children with community acquired severe pneumonia at Bugando Medical Centre, Mwanza, Tanzania: a prospective cohort study. PLoS One. (2020) 15(12):e0243636. 10.1371/Journal.Pone.0243636 33306722 PMC7732094

[25] Cherian T Mulholland EK Carlin JB Ostensen H Amin R Md C Standardized interpretation of paediatric chest radiographs for the diagnosis of pneumonia in epidemiological studies. WHO Bull. (2005) 83:353–9. PMC2626240 15976876

[26] Tsegaw H Yimam M Nureye D Woldeselassie W Hambisa S. Predictors of treatment outcomes among pediatric patients hospitalized with pneumonia in Tikur Anbessa Specialized Hospital, Addis Ababa, Ethiopia. Adv Pharmacol Pharm Sci. (2021) 2021:1–7. 10.1155/2021/6690622 PMC8079214 33987540

[27] Bekele F Sinaga M Quadri JA Kumar A Shariff A Malik T. Factors associated with outcomes of severe pneumonia in children aged 2 months to 59 months at Jimma University Specialized Hospital, Southwest Ethiopia. Curr Pediatr Res. (2017) 21(3):447–54.

[28] Dembele BPP Kamigaki T Dapat C Tamaki R Saito M Saito M Aetiology and risks factors associated with the fatal outcomes of childhood pneumonia among hospitalised children in the Philippines from 2008 to 2016: a case series study. BMJ Open. (2019) 9(3):e026895. 10.1136/Bmjopen-2018-026895 30928958 PMC6475207

[29] Jroundi I Mahraoui C Benmessaoud R Moraleda C Tligui H Seffar M Risk factors for a poor outcome among children admitted with clinically severe pneumonia to a university hospital in Rabat, Morocco. Int J Infect Dis. (2014) 28:164–70. 10.1016/j.ijid.2014.07.027 25305555 PMC7129557

[30] Gutu B Yoseph L Balisa M. Clinical treatment outcomes of pneumonia among hospitalized pediatric patients in Nekemte Referral Hospital, Pediatrics Ward, Ethiopia. World J Pharm Pharm Sci. (2017) 6(02):68–84.

[31] Zhu Z Zhang T Guo W Ling Y Tian J Xu Y. Clinical characteristics of refractory mycoplasma pneumoniae pneumonia in children treated with glucocorticoid pulse therapy. BMC Infect Dis. (2021) 21:1–8. 10.1186/s12879-020-05706-z 33509121 PMC7844890

[32] Theodoratou E Al-Jilaihawi S Woodward F Ferguson J Jhass A Balliet M The effect of case management on childhood pneumonia mortality in developing countries. Int J Epidemiol. (2010) 39(Suppl 1):i155–71. 10.1093/ije/dyq032 20348118 PMC2845871

[33] Tiewsoh K Lodha R Pandey RM Broor S Kalaivani M Kabra SK. Factors determining the outcome of children hospitalized with severe pneumonia. BMC Pediatr. (2009) 9:15. 10.1186/1471-2431-9-15 19236689 PMC2651138

[34] Salih KMA AliBilal J Karsani AH. Risk factors of mortality among children admitted with severe pneumonia at a reference hospital in Khartoum, Sudan. Am J Med Med Sci. (2015) 5(3):130–4.

[35] Morgan G. What, if any, is the effect of malnutrition on immunological competence? Lancet. (1997) 349(9066):1693–5. 10.1016/S0140-6736(96)12038-9 9186397

[36] Chisti MJ Tebruegge M La Vincente S Graham SM Duke T. Pneumonia in severely malnourished children in developing countries–mortality risk, aetiology and validity of WHO clinical signs: a systematic review. Trop Med Int Health. (2009) 14(10):1173–89. 10.1111/j.1365-3156.2009.02364.x 19772545

[37] Ferdous F Ahmed S Das SK Chisti MJ Nasrin D Kotloff KL Pneumonia mortality and healthcare utilization in young children in rural Bangladesh: a prospective verbal autopsy study. Trop Med Health. (2018) 46:17. 10.1186/s41182-018-0099-4 29875615 PMC5970515

[38] Dheresa M Dessie Y Negash B Balis B Getachew T Mamo Ayana G Child vaccination coverage, trends and predictors in Eastern Ethiopia: implication for sustainable development goals. J Multidiscip Healthc. (2021) 14:2657–67. 10.2147/JMDH.S325705 34584421 PMC8464587

[39] Ramachandran P Nedunchelian K Vengatesan A Suresh S. Risk factors for mortality in community acquired pneumonia among children aged 1–59 months admitted in a referral hospital. Indian Pediatr. (2012) 49(11):889–95. 10.1007/s13312-012-0221-3 22791667

